# *Candida auris* Bloodstream Infections in Russia

**DOI:** 10.3390/antibiotics9090557

**Published:** 2020-08-30

**Authors:** Natalia E. Barantsevich, Antonina V. Vetokhina, Natalia I. Ayushinova, Olga E. Orlova, Elena P. Barantsevich

**Affiliations:** Almazov National Medical Research Centre, 2 Akkuratova str., 197341 St. Petersburg, Russia; natabara@mail.ru (N.E.B.); an.vetohina@yandex.ru (A.V.V.); katnatlove@mail.ru (N.I.A.); o.e.orlova@yandex.ru (O.E.O.)

**Keywords:** treatment, echinocandins, azoles, susceptibility, amphotericin B, mortality, attributable mortality, risk factors, K143R substitution, *EGR11* gene, phylogenetic analysis

## Abstract

*Candida auris*—a fungus (yeast) that can cause hospital outbreaks was first recognized in 2009. The authors report data on 38 cases of *C. auris* bloodstream infections in multidisciplinary hospitals situated in two distantly located regions of Russia, considering predisposing factors, antifungal susceptibility of isolates, treatment, and outcomes. Interhospital transfers of patients and labor migration contributed to the spread of *C. auris*. The South Asian lineage of the studied strains was indicated by K143R substitution in *ERG11* gene and phylogenetic analysis of internal transcribed spacer and D1-D2 domain. All isolates from *C. auris* candidemia cases were susceptible to echinocandins. High-level resistance to fluconazole and resistance to amphotericin B were present in the majority of strains. The overall all-cause mortality rate in *C. auris* bloodstream infections was 55.3% and the 30-day all-cause mortality rate 39.5%. The attributable mortality was 0%. Eradication of *C. auris* from blood was associated with the favourable outcomes in patients. It was achieved irrespective of whether antifungal preparations within or outside the susceptibility range were administered. Further international surveillance and studies providing consensus guidelines for the management of *C. auris* infections are needed.

## 1. Introduction

*Candida auris* was first recognised as a novel *Candida* species in 2009 when it was isolated from an ear discharge of an inpatient—a Japanese woman 70 years of age at Tokyo Metropolitan Geriatric Hospital [1,2]. The first three cases of nosocomial bloodstream infections, caused by the fungus were reported from South Korea in 2011 with one strain, isolated but not identified in 1996 [3]. Another *C. auris* isolate from blood, unidentified in 2008 in Pakistan, was later reclassified as *C. auris* [2,4]. Only a few isolates of the fungus were found by retrospective analysis of tens of thousands *Candida* strains from various collections [2]. Whole genome sequencing of *C. auris* strains, isolated in different parts of the world demonstrated that the spread of the fungus did not start in a single place, but, more likely, in at least four locations independently forming four distinct clades with isolates separated by more than 70,000 single nucleotide polymorphisms (SNPs). These clades were South Asian (Clade I), East Asian (Clade II), African (Clade III) and South American (Clade IV) lineages [4,5]. The strains within the clades appeared to be clonal [4]. Recently a fifth clade of *C. auris* was discovered in Iran in a patient, who had never been outside the country. The isolate had more than 200,000 SNPs difference with all known clades [6]. A possible explanation of an almost simultaneous spread of the yeast in different parts of the world could be an increase in the selective pressure of widely used antifungals in agriculture, humans and animals [4]. Another possible explanation is the development of *C. auris* into human pathogen as an impact of a current climate change that allowed the fungus to adapt to the temperature of a human body and cause infections [7].

*C. auris* has been isolated in more than 30 countries on six continents [8]. The fungus is currently under international surveillance [9]. Antimicrobial resistance to multiple widely used systemic antifungals with the potential to contaminate hospital environments, and with resistance to a wide range of disinfectants, ability to cause bloodstream infections in critically-ill patients and outbreaks in the intensive care units (ICU) lead to the recognition of *C. auris* as one of the most important pathogens, that are currently emerging [10].

## 2. Results

*C. auris* bloodstream infections diagnosed in 38 in-patients from two multidisciplinary public hospitals situated in two distantly located regions of Russia in 2017–2019 were included in the study. The first hospital was situated in the Central region, the second in the Siberian region. All patients were in the ICU at the time of the first isolation of the fungus from blood. The mean time of the ICU stay preceding the onset of *C. auris* bloodstream infection was 39 ± 4, 8 days (range 1–86 days).

The index case of *C. auris* candidemia in the first hospital occurred in March 2017 in a patient with preceding *C. auris* urinary tract infection. *C. auris* was isolated for the first time in the same ICU five months earlier from a urine sample of a labour migrant from Tajikistan, colonised with the fungus. The index case of *C. auris* bloodstream infection in the second hospital was in a patient with already existing *C. auris* candidemia transferred to the ICU in February 2018 from a public hospital located in a small town of the same region. The patient did not have international travel history.

The mean age of patients was 55.0 ± 4.7 years (range 15–87 years), 28 patients were male, 10 were female. The underlying conditions included severe multitrauma in 23 (60.5%), pancreatic necrosis in four (10.5%), abdominal surgery in four (10.5%), brain injury in three (7.9%), community acquired pneumonia in two (5.3%), and heart or liver failure in two (5.3%) cases. Major surgical operations were performed in 34 (89.5%) patients. Sepsis and bacterial bloodstream infections prior or concurrently with *C. auris* bloodstream infection were diagnosed in 36 (95.0%) patients. They preceded isolation of *C. auris* from blood in 31 (82.0%) cases and were observed at the time of *C. auris* bloodstream infection in 16 (42.1%) patients. Nosocomial bacterial pneumonia was present in 29 (76.3%) cases before and in 25 (65.8%) cases concurrently with *C. auris* bloodstream infection. Infections, caused by other *Candida* spp. (*C. albicans*, *C. glabrata*, *C. parapsilosis*, *C. krusei*, *C. tropicalis*) were present during or prior to *C. auris* bloodstream infections in 24 (63.2%) cases. *C. auris* urinary tract infection preceded isolation of the fungus from blood in 13 (34.2%) patients. Impairment of glucose metabolism (diabetes mellitus and/or stress hyperglycemia) were present in 22 (57.9%) patients, corticosteroids were administered in 7 (18.4%) cases. All patients received broad spectrum antibiotics prior to *C. auris* isolation from blood. Administration of antifungal preparations (fluconazole in daily dose 150 mg during 27 ± 4.8 days in 20 cases, voriconazole in daily dose 200 mg during seven days in one case) for different conditions preceded the first isolation of *C. auris* from blood in 21 (55.3%) patients. All patients had central venous and urine catheters.

Minimum inhibitory concentrations (MICs) of systemic antifungal preparations were determined in non-duplicate isolates (Table 1).

High level resistance to fluconazole (MIC ≥ 256 mg/L) was revealed in 37 (97.4%) strains, resistance to amphotericin B with MIC ≥ 2 mg/L in 29 (76.3%) isolates. All strains were susceptible to echinocandins.

*C. auris* bloodstream infections were treated with echinocandins in nine cases (the strains had MIC 0.25–0.5 mg/L): five patients received caspofungin in daily dose 50 mg after loading dose 70 mg during seven days, and three patients were treated with micafungin in daily dose 150 mg during 3–10 days. Eradication of *C. auris* was achieved in five (55.6%) patients, and three of them survived. The overall all-cause mortality rate in patients treated with echinocandins was 66.7%**,** the 30-day all-cause mortality rate was 55.6%, the attributable mortality was 0%. The growth of multiple bacterial species, but not *C. auris* was detected in the postmortem study of internal organs.

Fluconazole was administered in 25 cases with high level resistance to fluconazole (MIC ≥ 256 mg/L). The overall mortality rate in these patients was 52.0%, the 30-day all-cause mortality rate 36.0%. The attributable mortality was 0%. The postmortem microbiological study of internal organs was performed in two cases and did not reveal *C. auris*, various bacterial species were cultured. MIC of fluconazole in one patient was 4 mg/L. The patient had two episodes of *C. auris* candidemia, treated with fluconazole. She received the preparation in daily dose 150 mg for 25 days and 15 days. Though the fungus was eradicated from blood in both episodes, unfortunately the patient died.

Voriconazole was administered to one patient in daily dose 200 mg for seven days, and the strain had MIC 2 mg/L. *C. auris* was eradicated from blood, the patient survived.

One patient received amphotericin B in daily dose 12.5 mg during 10 days. The MIC of amphotericin B was >8 mg/L, and the isolate was considered resistant. The fungus was eradicated from blood, the patient survived.

The overall all-cause mortality rate was 70.0% in 10 patients treated with agents within susceptibility range—echinocandins (9 patients) or fluconazole (one patient). It was 50.0% in 26 patients who received fluconazole (25 patients) or amphotericin B (one patient)—preparations outside the susceptibility range (*p* = 0.279401).

The overall all-cause mortality in *C. auris* bloodstream infections was 55.3%, the 30-day all-cause mortality 39.5%, the attributable mortality 0%.

*C. auris* was eradicated from blood in 21 (55.3%) cases. The overall mortality rate in patients with achieved eradication was 33.3%, in patients without eradication 82.4%. (*p* = 0.002513).

Eradication of *C. auris* from blood was achieved in 60.0% patients treated with preparations within susceptibility range (echinocandins, fluconazole) and in 53.8% cases with administered agents outside the susceptibility range (fluconazole, amphotericin B), *p* = 0. 73927.

**Figure 1:**
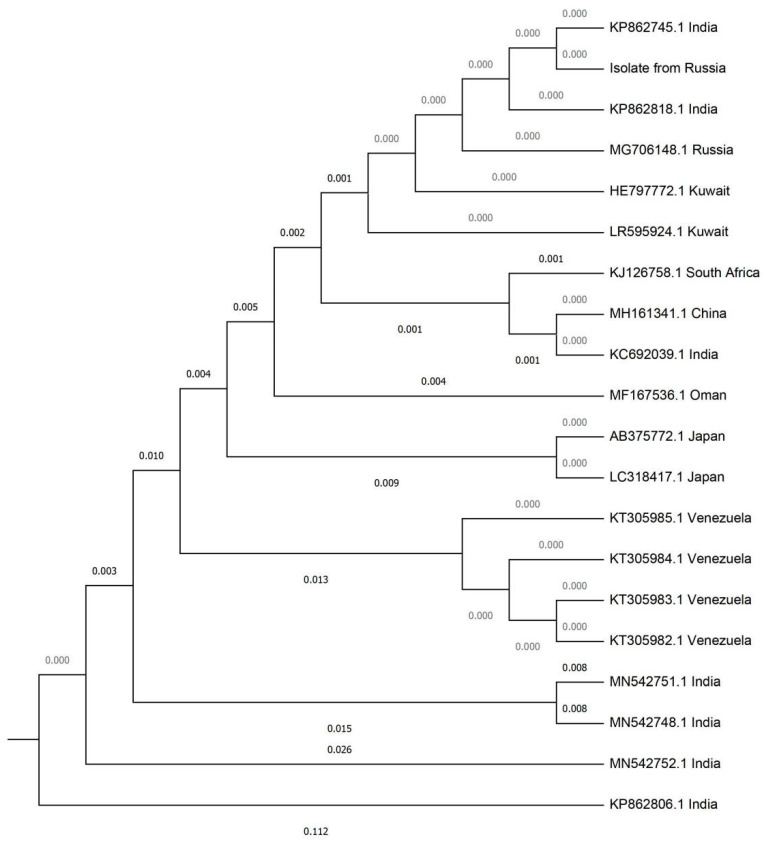
Phylogenetic tree of ITS region.

Genetic studies were performed in 11 available strains (seven isolates from the first hospital and four isolates from the second hospital). All isolates had identical nucleotide sequences in internal transcribed spacer (ITS) and D1-D2 regions. Phylogenetic analysis of the ITS region revealed that the isolates clustered with strains from India, Kuwait and Russia of South Asian clade (Figure 1).

**Figure 2:**
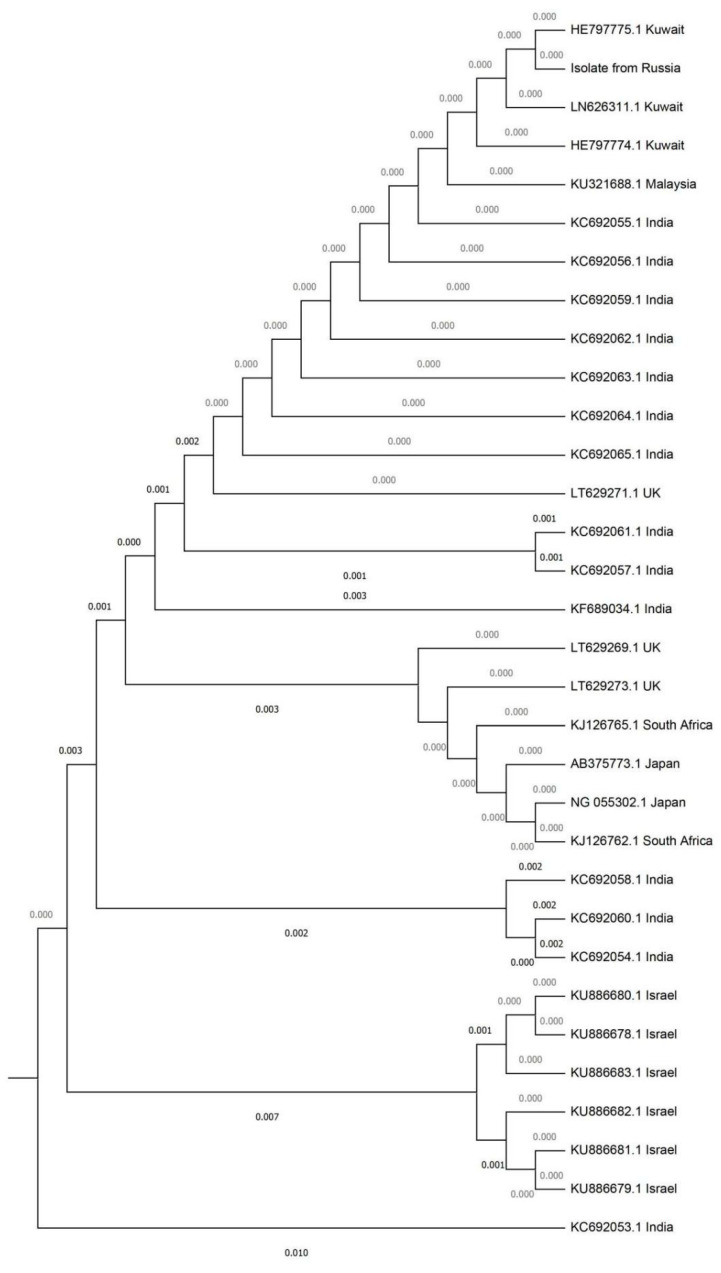
Phylogenetic tree of D1-D2 domain.

The phylogenetic analysis of D1-D2 domain demonstrated clustering of studied strains with isolates from India, Kuwait, Malaysia, and the UK of South Asian lineage (Figure 2).

All studied isolates had identical nucleotide sequences with K143R substitution in the *ERG11* gene. The *ERG11* gene as well as ITS region had 100% identity with the strain from South Asian clade isolated from a patient at a Moscow hospital in October 2017 (GenBank accession numbers MG706148.1, SBIO00000000.1).

## 3. Discussion

*C. auris* is an emerging fungal nosocomial pathogen that has been encountered in dozens of countries on 6 continents a decade since its first discovery. Several factors are known to contribute to the spread of nosocomial pathogens between countries and hospitals: overseas travel and medical care, migration, interhospital transfers of patients etc. [11,12,13,14,15]. Two of them were associated with the introduction of the fungus into two hospitals in distantly located regions in Russia: labour migration and interhospital transfers. The known risk factors for *C. auris* infections were present in our patients: all patients received broad spectrum antibiotics prior to candidemia, all had indwelling catheters (venous and urinary), the majority of patients had underwent major surgery, had impaired glucose metabolism and/or received systemic antifungals [16]. The additional not widely recognised risk factors for *C. auris* candidemia in our series were other *Candida* infections (*C. auris* urinary tract infection and/or various infections caused by other *Candida* spp.) and/or severe bacterial infections (prior and/or concurrently). They were present in all our patients. The severe bacterial infections (sepsis and pneumonia) had a clear impact on the patient’s outcomes and were found at postmortem examination contrary to *C. auris*. The role of other infections in patients with *C. auris* candidemia to our estimation is currently poorly understood and rarely reported. The polymicrobial (bacterial and fungal) infections in our series were observed in patients with severe underlying conditions the majority had high-energy trauma and preceding major surgery often with unfavourable prognosis. There is a need for further studies in patients with less severe underlying conditions to understand whether other infections should be considered as a factor predisposing to or affecting the outcome in patients with *C. auris* candidemia.

The overall all-cause mortality rate in the reported cases of C. auris candidemia was 29–72%. The 30-day all-cause mortality in the outbreak in Spain was 41.4%, in the series from India 41.9% [10,17,18,19,20,21,22]. The overall all-cause mortality in our series was 55.3%, the 30-day all-cause mortality 39.5%. The attributable mortality was 0%. The similar results with no attributable mortality in eight patients with *C. auris* candidemia were reported in the first outbreak in London [23]. The mortality rates in our series were consistent with the previously reported data, but obtained in the same group of patients clearly demonstrated discrepancy in all-cause and attributable mortality posing question on the exact impact of *C. auris* bloodstream infection on the patients’ conditions and outcomes.

There is an important problem concerning *C. auris* bloodstream infections regarding what management should be considered appropriate and how to choose the right medication. The fungus has only one copy of the *ERG3* and *ERG11* genes, as well as the *FKS1*, *FKS2* and *FKS3* genes associated with resistance to azoles and echinocandins respectively [24]. There is therefore a possibility of a rapid emergence and spread of multidrug- and pan-drug resistant strains in case of widespread use of these agents, especially taking into account the frequent resistance of the fungus to amphotericin B. Pan-drug resistant strains of *C. auris* have already been encountered [25,26]. There is currently not enough evidence of the performance of systemic antifungals in *C. auris* bloodstream infections due to different underlying conditions in still limited number of reported cases and different approaches to analysis of mortality rate [22,27]. The unresolved problems with the choice of medications in *C. auris* candidemia may contribute to insufficient results. First, the breakpoints for *C. auris* have not been established either by Clinical and laboratory standards institute (CLSI) or European Union committee on antimicrobial susceptibility testing (EUCAST) until now. There are possibilities to follow recommendations of the Center for disease control and prevention (CDC) for a limited number of agents (amphotericin B, fluconazole and echinocandins) or to use *C. albicans* (or other *Candida spp.*) breakpoints as a substitution for *C. auris* [2,4,24,28,29]. The latter presents additional uncertainty due to the discrepancy of CLSI and EUCAST breakpoints for *C. albicans*. Therefore the CDC recommendations were used in our study for assessment of susceptibility of isolates to antifungals. Second, another problem is the lack of international consensus guidelines and, in many countries, including Russia, national regulations on the treatment of *C. auris* bloodstream infections. Third, CDC recommends use of echinocandins, to which *C. auris* demonstrated susceptibility in the majority of the studied strains as first line, or lipid formulations of amphotericin B, but does not strictly limit the use of other antifungals [30]. In our series, echinocandins did not perform superior to fluconazole, the agent with high-level resistance in the majority of strains. All the facts mentioned above indicate the need for further studies of the treatment approaches in critically ill patients with *C. auris* and prove every single case of candidemia to be a valuable entity for further international consensus on the management of *C. auris* bloodstream infections.

The important finding in our series was the effect of eradication of *C. auris* from blood that was associated with favourable outcomes in patients. Eradication of *C. auris* from blood was achieved irrespective to whether the fungus was susceptible or resistant to the administered preparation. This fact additionally stresses the need for further studies and assessment of different approaches to the management of *C. auris* candidemia in patients with various underlying conditions.

All studied isolates shared 100% nucleotide identity for ITS region, D1-D2 domain, and *ERG11* gene. They had K143R mutations in *ERG11* gene typical to Clade I. The strain of the same lineage caused bloodstream infection in a Moscow hospital in October, 2017, thus suggesting the clonal spread of South Asian clade in Russia [16,31].

## 4. Materials and Methods

Cases of *C. auris* bloodstream infection from two multidisciplinary public hospitals were included in the study. These hospitals were situated approximately 5200 km apart in the cities located in two different regions of Russia: the Central Federal District (the first hospital) and the Siberian Federal District (the second hospital).

The study was carried out prospectively and retrospectively. All laboratory studies, except the genetic studies of fungi, were performed prospectively. The authors did not influence in any aspect the choice of medications for the treatment of *C. auris* bloodstream infections or any other conditions and diseases. The choice of preparations was made by the physician and/or clinical pharmacist as required by the regulations in Russia. The results of the laboratory findings were reported to the physicians immediately.

### 4.1. Samples Collection and Processing

Blood samples from inpatients of the first hospital were inoculated into aerobic blood culture bottles in 10 mL volume. The bottles were incubated in BACTEC FX400 (Becton, Dickinson and Company, Franklin Lakes, NJ, USA) at 37 °C until growth was detected. After the presence of fungi was confirmed microscopically, the samples were cultured on chromogenic agar (Liofilchem, Roseto degli Abruzzi, Italy) and Sabouraud dextrose agar (Oxoid, Basingstoke, UK) for 24–48 h. Identification to species level was performed with MALDI-TOF mass-spectrometry (MicroFlex LT/SH, Bruker Daltonics Inc, Billerica, MA, USA) and MALDI Biotyper 3.1 software with MBT 8468 MSP Library database (Bruker Daltonics Inc., Billerica, MA, USA), according to manufacturers’ manual.

Blood samples from inpatients of the second hospital were inoculated into aerobic blood culture bottles in 10 mL volume. The bottles were incubated in BactAlert 120 (Biomerieux, Marcy l’Etoile, France) at 37 °C until growth was detected. After the presence of fungi was confirmed by microscopic examination, the samples were plated on chromogenic Brilliance Candida agar (Oxoid, Basingstoke, UK) and Sabouraud dextrose agar (Oxoid, Basingstoke, UK). Identification to species level was performed with Vitek MS MALDI-TOF mass spectrometer (bioMérieux, Marcy l’Etoile, France) and VITEK MS V3.2 database (bioMérieux, Marcy l’Etoile, France) according to manufacturer’s manual.

MICs of systemic antifungal agents: amphotericin B, 5-fluorocytosine, azoles (fluconazole, voriconazole, itraconazole, posaconazole), and echinocandins (anidulafungin, caspofungin and micafungin) were studied with Sensititre YeastOne AST plates (Thermo Fisher Scientific, Waltham, MA, USA) in both hospitals. The results were analyzed after 24 h incubation of plates at 35 °C. The change in colour of medium and the inhibition of growth were considered according to the manufacturer’s instructions. CDC tentative breakpoints for *C. auris* were used for the evaluation of the results [31]. Isolates with MIC ≥ 32 mg/L for fluconazole and ≥2 mg/L for amphotericin B were considered resistant. Susceptibility to echinocandins was registered with MIC of anidulafungin <4mg/L, caspofungin <2 mg/L, micafungin <4 mg/L.

The attributable mortality was evaluated by the independent forensic center for patients with lethal outcomes from the first hospital and by the independent pathology center in patients with lethal outcomes from the second hospital. The microbiological study of the internal organs and tissues from patients of the second hospital was additionally performed in the microbiological laboratory of the same hospital. Approximately 5 g of spleen, heart tissue and other tissues according to the localization of pathologic abnormalities were dissected using sterile scalpels and placed in polypropylene sterile containers in an examination area. The samples were transported from the pathology center immediately to the microbiological laboratory of the second hospital. The samples were additionally dissected in sterile conditions and 0.5 g sections were incubated in 4.5 mL of the Sabouraud dextrose broth for 24–48 h and subcultured into Sabouraud dextrose agar (Oxoid, Basingstoke, UK), Candida agar (Oxoid, Basingstoke, UK), blood agar, and incubated at 37 °C for 24–48 h. The identification of microorganisms was performed with VitekMS (Biomerieux, Marcy l’Etoile, France) and VITEK MS V3.2 database (Biomerieux, Marcy l’Etoile, France).

### 4.2. Genetic Studies

The genetic studies included Sanger sequencing of ITS region and D1-D2 domain of the ribosomal DNA of *C. auris* isolates. The same protocol for both reactions was used. PrepmanUltra (Thermo Fisher Scientific, Waltham, MA, USA) was used for DNA extraction, according to the manufacturers’ manual. Working DNA solution was prepared by addition of deionized water to supernatant, in 50:1 ratio. PCR Master mix was prepared, using AmpliTaq Gold Master Mix (Thermo Fisher Scientific, Waltham, MA, USA) according to the manufacturers manual. PCR reaction was performed, using the following protocol: initial denaturation at 95 °C for 10 min followed by 35 cycles: denaturation at 94 °C for 1 min, annealing at 55 °C for 1 min and extension at 72 °C for 1.5 min. The final cycle was followed by elongation at 72 C for 10 min. The following primers were used for amplification: ITS-1 and ITS-4 for ITS region, NL-1 and NL-4 for D1/D2 region (Table 2) [32,33].

PCR product was purified, using EDTA/ethanol precipitation. Sequencing reaction mix was prepared with the use of BigDye Terminator v1.1 Cycle Sequencing Kit (Thermo Fisher Scientific, Waltham, MA, USA) according to the manufacturer’s manual. Sequencing was performed with ABI 3130 Genetic Analyser (Thermo Fisher Scientific, Waltham, MA, USA) according to the manufacturer’s instruction. Results were analyzed with Sequencing Analysis v.5.3.1. program. Homologous sequences were searched for in GenBank, National Center for Biotechnology Information (NCBI) database. with the use of BLAST program. The sequences were aligned with ClustalW alignment. Phylogenetic analysis was performed in MEGA X program.

Study of *ERG11* gene of *C. auris* isolates was performed with primers, which design was based on the sequence, obtained from NCBI database, GenBank accession no. KY410388.1 as reported previously [16]. Totally 3 primer sets were used for amplification and sequencing of *ERG11* gene of *C.auris* isolates (Table 2). The following conditions were applied: 5 min initial denaturation at 95 °C followed by 30 cycles (30 s at 95 °C, 30 s at 56 °C, 90 s at 72 °C). The final elongation was carried out for 7 s at 72 °C The sequencing was performed with the same primers and BigDye Terminator Kit v1.1 (Applied Biosystems, USA) and ABI 3130 Genetic Analyser (Applied Biosystems, Beverly, MA USA) according to the manufacturer’s manual. Sequences were analysed and aligned with sequences from GenBank with ClustalW alignment.

### 4.3. Statistical Analysis

Pearson’s χ^2^ test was used for categorical variables in statistical analysis. The result was considered significant at *p* < 0.05.

## 5. Conclusions

The spread of the emerging nosocomial pathogen *C. auris* to two different regions in Russia was associated with interhospital transfers of patients and labour migration. Well-known risk factors (administration of antibiotics and antifungals, indwelling catheters, operations, impaired glucose metabolism) were present in our study. The underestimated risk factors for *C. auris* bloodstream infections were severe bacterial infections (sepsis, pneumonia) and other *Candida* infections observed in all studied cases. The majority of *C. auris* strains had high-level resistance to fluconazole. They were resistant to amphotericin B and susceptible to echinocandins. All studied isolates had identical nucleotide sequences in ITS and D1-D2 regions and clustered with strains of Clade 1. *ERG11* gene had K143R substitution typical to South Asian lineage. The outcomes in patients with *C. auris* bloodstream infections treated with echinocandins and in patients who received fluconazole and had strains with high level resistance to this agent were similar. The eradication of *C. auris* from blood correlated with the favourable outcomes and was achieved with agents within and outside the susceptibility range. These data as well as discrepancies in high overall all-cause mortality (55.3%) and attributable (0%) mortality clearly indicated the need for further international surveillance and studies, especially taking into account the current lack of international consensus guidelines for the management of *C. auris* bloodstream infections and established both CLSI and EUCAST breakpoints for the fungus.

## Figures and Tables

**Table 1:** Minimum inhibitory concentrations results for *C. auris* isolates.

Antifungal Preparation	MIC, Range (mg/L)	MIC_90_ (mg/L)	MIC_50_ (mg/L)
Amphotericin B	≤0.12–>8	4	2
5-Fluorocytosine	≤0.06–0.5	0.25	0.12
Fluconazole	4–>256	>256	>256
Voriconazole	0.12–8	4	2
Itraconazole	0.06–2	0.5	0.5
Posaconazole	0.03–1	0.5	0.25
Anidulafungin	0.03–1	0.5	0.25
Micafungin	0.03–1	0.25	0.12
Caspofungin	0.06–1	0.5	0.5

**Table 2:** Primers used for amplification.

Gene/Region	Primer	Oligonucleotides
ITS	ITS-1	TCCGTAGGTGAACCTGCGG
ITS	ITS-4	TCCTCCGCTTATTGATATGC
D1-D2	NL-1	GCATATCAATAAGCGGAGGAAAAG
D1-D2	NL-4	GGTCCGTGTTTCAAGACGG
*ERG11*	1F	TCTCAGAAAAGACAGAGCTC
*ERG11*	1R	CTTCACGCCATCTTTATACG
*ERG11*	2F	GTTAGGAAAAGTTATGACGG
*ERG11*	2R	TTGGTGACTTTACCAAACCC
*ERG11*	3F	AGATCTCTGCTACCTACATG
*ERG11*	3R	GATTTCTGCTGGCTCCATTG
